# Hepatothorax after right-sided diaphragmatic rupture mimicking a pleural effusion: a case report

**DOI:** 10.4076/1757-1626-2-8545

**Published:** 2009-07-14

**Authors:** Franz Georg Bader, Martin Hoffmann, Tilman Laubert, Uwe Johannes Roblick, Andreas Paech, Hans-Peter Bruch, Lutz Mirow

**Affiliations:** 1 Department of Surgery and Trauma, University of Schleswig-HolsteinCampus Lübeck, Ratzeburger Allee 160, 23538 LübeckGermany; 2 Department of Orthopedics and Trauma, University of Schleswig-HolsteinCampus Lübeck, Ratzeburger Allee 160, 23538 LübeckGermany

## Abstract

**Introduction:**

Diaphragmatic ruptures are a rare condition with an incidence of about 0.8-5.8% after blunt thoracoabdominal trauma. Right sided ruptures accompanied by a displacement of intraabdominal organs are very uncommon and account for approximately 5-19% of all diaphragmatic ruptures. The majority of diaphragmatic ruptures are based on high speed motor vehicle accidents (MVA) and high falls.

**Case presentation:**

Herein we report a case of a 58-year old woman after a high-speed MVA with a right-sided diaphragmatic rupture and displacement of the liver into the thorax, mimicking a pleural effusion.

**Conclusion:**

Due to the low incidence and frequently present masking injuries, diagnosis is difficult and virtually always delayed. Thus, a high index of suspicion is important in cases of blunt thoracoabdominal trauma, as the 24 h mortality-rate of a right sided diaphragmatic rupture is up to 30%. In these situations a spiral CT-scan is the diagnostic tool of choice. Surgical intervention using an abdominal approach via a hockey-stick shaped incision is necessary even for small tears. Part of the polytrauma management following high speed MVAs is a critical review of the radiologic imaging.

## Introduction

Diaphragmatic ruptures are a rare condition with an incidence of about 0.8-5.8% after blunt thoracoabdominal trauma [1-3]. The majority of diaphragmatic ruptures are based on high speed motor vehicle accidents (MVA) and high falls. Right sided ruptures accompanied by a displacement of intraabdominal organs are compared to left sided ruptures very uncommon and account for approximately 5-19% of all diaphragmatic ruptures [1,3,4]. Due to the low incidence and frequently present masking injuries, diagnosis is difficult and virtually always delayed. Part of the polytrauma management following high speed MVAs is a critical review of the radiologic imaging. A high index of suspicion is important in cases of blunt thoracoabdominal trauma, as the 24 h mortality rate of a right sided diaphragmatic rupture is up to 30% [1,3,5]. We present a case of a right sided diaphragmatic rupture with herniation of the entire right hepatic lobe into the thorax after a high-speed MVA, initially misinterpreted as a pleural effusion.

## Case presentation

After a high speed MVA, a 58-year old white German woman was initially admitted to a primary care hospital in northern Germany. She was conscious and accessible but suffered from severe pain in the legs and the pelvis as well as from acute dyspnea. The initial diagnostic work-up including spiral-CT-scan revealed multiple fractures of the upper and lower extremities as well as a right sided posterior wall acetabular fracture accompanied by fractures of the superior and inferior pubic bone. Due to an assumed massive right sided pleural effusion a thoracic tube was introduced. The patient was then transferred after initial hemodynamic stabilization to the Department of Surgery, University of Schleswig-Holstein, Campus Lübeck, Germany.

**Figure 1.**
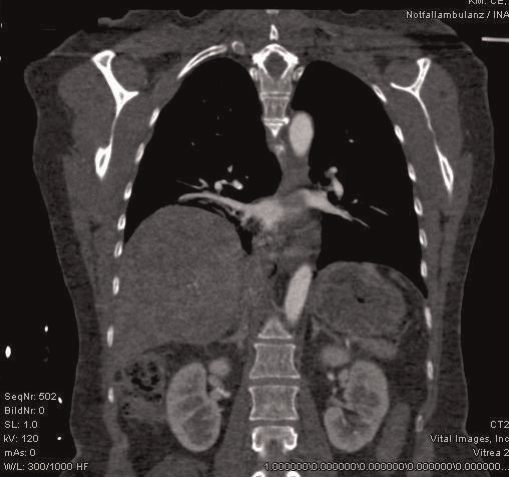
Reconstruction of the CT-scan: full displacement of the liver into the right thorax.

On admission, the patient was still haemodynamically stable. There was no drainage from the thoracic tube. The routine review of the provided copies of the spiral CT-scan showed a displacement of large parts of the liver into the right hemithorax. Moreover, the superior vena cava was compacted (Figure 1).

The torsion of the superior vena cava and the displacement of large parts of the liver consecutively resulted in worsening of breathing abilities with corresponding elevation of pCO_2_ as well as tachycardia and hypotension. The patient was immediately transferred to the operating room without further delay.

A hockey-stick shaped laparotomy was performed. There were no further damages to other intra-abdominal organs or structures.

Intraoperatively we detected a right sided diaphragmatic rupture encountering the entire right lobe of the liver herniating into the right hemithorax. We performed a complete mobilization of the liver. After this maneuver we were able to relocate the liver into the abdomen. Throughout this part of the operation meticulous care was taken on the preservation of the vena cava.

Careful inspection of the liver revealed a superficial scrab over the segments VI and VII where the drainage has been placed into the thorax.

The diaphragm was closed with intermittent stitches. The defect area was then augmented with a polypropylene mesh, fixed onto the inferior surface of the diaphragm. Immediately after replacement of the liver, the hemodynamic and pulmonary situation stabilized. There were no further thoracic or abdominal complications during hospital stay.

## Discussion

A traumatic diaphragmatic rupture is an unusual complication of blunt or penetrating thoracoabdominal trauma [6]. The most common classification by Strug and colleagues classifies the diaphragmatic ruptures as Type I (immediate onset of clinical symptoms) and Type II (late onset of symptoms) [7]. Right sided ruptures of the diaphragm are compared to left sided ruptures a rare disease entity with an incidence of 5-19% of all diaphragmatic ruptures [8,9]. As in the case presented here it is most frequently connected to a high-velocity blunt or penetrating trauma to the abdomen and/or thorax [3,8].

The incidence of herniations following diaphragmatic rupture is about 19% for right sided ruptures, while it is 58% for left sided ruptures [1,3]. An explanation might be a protective effect of the liver preventing most herniations.

Most patients suffer from dyspnea and pain in the upper abdomen and lower thorax. Other commonly described clinical presentations include cyanosis, cardiac arrhythmia and hypotension. Injuries that should prompt suspicion for an accompanying rupture of the diaphragma include pericostal injuries, fractures of the pelvis and lumbar spine, auscultation of bowel sounds in the chest and dullness on percussion of the thorax.

In most institutions, the initial diagnostic tool is still the conventional chest X-ray. This technique has certain limitations concerning sensitivity and specificity as only 17-40% of patients are correctly diagnosed by this imaging technique [10-12]. A CT-scan is of superior diagnostic value with a specificity of nearly 100% and a sensitivity of 50% for right sided ruptures [13]. Moreover, a thoracoabdominal spiral CT-scan offers additional information and should be implemented in such cases.

The repair of the diaphragma was done via a hockey stick shaped incision starting below the xiphoid and then bending to the right continuing infracostally. This offers excellent overview of the abdominal cavity. A complete exploration of all organs and the retroperitoneum is possible to rule out any further injuries.

In the situation of an acute traumatic rupture we would therefore argue for this approach. In case of a chronic herniation of intraabdominal organs into the thorax an abdominothoracic or thoracic approach might be necessary [1,3,6].

From our point of view this case is special because of the initial misinterpretation of the spiral-CT-scan and introduction of a thoracic tube into the right hemithorax to drain an inexisting pleural effusion. Therefore, a careful examination of the existing radiologic imaging is mandatory.

The current literature provides some reports dealing with right sided diaphragmatic rupture accompanied by herniation of the liver into the thorax. Notably, the majority report delayed diagnosis [2,4,14,15]. Thus, a high index of suspicion is of paramount importance for the diagnosis of diaphragmatic ruptures in any patient after high velocity blunt thoracoabdominal trauma.

## Conclusion

In patients with blunt thoracoabdominal trauma and/or high-velocity injuries a high rate of suspicion for a diaphragmatic rupture is of paramount importance. In these situations a spiral CT-scan is the diagnostic tool of choice. Surgical intervention using an abdominal approach via a hockey-stick shaped incision is necessary even for small tears. Delayed diagnosis is common, predicting the high mortality-rate of 30% [1,3,5,15].

## Abbreviations

CT: Computed tomography
MVA: Motor vehicle accidents
